# Point-of-Care Ultrasound Identifies Decompensated Heart Failure in a Young Male with Methamphetamine-Associated Cardiomyopathy Presenting in Severe Sepsis to the Emergency Department

**DOI:** 10.1155/2018/2859676

**Published:** 2018-10-09

**Authors:** David Kinas, Michael Dalley, Kayla Guidry, Mark A. Newberry, David A. Farcy

**Affiliations:** ^1^Mount Sinai Medical Center, 4300 Alton rd., Miami Beach, FL 33140, USA; ^2^FIU Herbert Wertheim College of Medicine, 1120 SW 8th st., Miami, FL 33199, USA

## Abstract

We describe a case of a young male who presents to the emergency department with severe sepsis and decompensated heart failure with underlying Methamphetamine-Associated Cardiomyopathy that was previously undiagnosed. This presentation is unique because Methamphetamine-Associated Cardiomyopathy is an uncommonly reported condition that presented in a complex clinical scenario of severe sepsis and decompensated congestive heart failure. We discuss how we used point-of-care ultrasound (POCUS) in this case to identify an unsuspected disease process and how it changed our initial resuscitation strategy and management. Emergency physicians can utilize point-of-care ultrasound (POCUS) to help identify these high-risk patients in the emergency department and guide appropriate resuscitation. Methamphetamine-Associated Cardiomyopathy (MAC) is an infrequently described complication of methamphetamine abuse, most commonly presented as a nonischemic dilated cardiomyopathy. With the rise in methamphetamine abuse in the United States, complications from methamphetamine use are more commonly presenting to the emergency department. Proper education and rehabilitation, with a goal of abstinence from amphetamine use, may allow patients to potentially regain normal cardiac function. Since the majority of patients present late with severe cardiac dysfunction, early detection is essential amongst critically ill patients since recognition may significantly influence ED management.

## 1. Introduction

Methamphetamine-Associated Cardiomyopathy (MAC) is an infrequently described complication of methamphetamine abuse which has a significant impact on morbidity amongst users. With the rise in methamphetamine abuse in the United States, emergency physicians should be alert to this condition. Proper education and rehabilitation, with a goal of abstinence from amphetamine use, may allow patients to potentially regain normal cardiac function. Additionally, early detection is essential amongst patients who present critically ill since recognition may impact the resuscitation strategy. Point-of-care ultrasound (POCUS) has been shown to change diagnosis, treatment plans, and dispositions when utilized in the emergency department. We describe a case of a young male who presents to the emergency department with previously undiagnosed Methamphetamine-Associated Cardiomyopathy (MAC) complicated by severe sepsis secondary to Influenza B, pneumonia, and bacteremia. Point-of-care ultrasound (POCUS) prompted early identification of an unsuspected dilated cardiomyopathy with concomitant congestive heart failure which had major impact on our initial resuscitation strategy and management.

## 2. Case Report

A 34-year-old male presented to our Emergency Department via emergency medical services complaining of palpitations, shortness of breath, and cough with one episode of hemoptysis that started earlier that day. The patient reported that his symptoms rapidly progressed a few hours prior to arrival shortly after smoking crystal methamphetamine. The patient reported chronic methamphetamine use for 10 years, becoming an everyday user 3 years ago. He admitted to occasional polysubstance use that included ketamine, ecstasy, and alcohol, although those were limited to a few times a month. Past medical history was significant for two remote spontaneous pneumothoraces, but the patient stated that his current symptoms felt unrelated. Past surgical history was noncontributory and he denied allergies or taking any prescribed medications. Reviews of systems were positive for myalgias and fatigue. The patient denied any chest pain, chest tightness or abdominal pain.

Initial vital signs include the following: HR: 153 bpm, RR: 20, BP: 115/56 mmHg, Temp (O): 102.1 °F, and SpO2 96% (RA).

The physical exam demonstrated a well-nourished, well-kept male appearing in his stated age in good physical shape. The patient appeared anxious and diaphoretic with dry mucus membranes and labored breathing. The patient was alert and oriented but found to have mild confusion upon detailed questioning. The rest of the neurologic exam was normal and the patient did not display any meningeal signs. Auscultation of the chest revealed rales at the lung bases. Cardiac exam demonstrated a regular, tachycardic rhythm without the presence of obvious murmurs, rubs, or gallops. There was no lower extremity edema present and patient displayed normal muscle tone. The rest of the physical exam was unremarkable.

Upon the patient's initial presentation, orders for initial management were with a normal saline bolus of 2 liters, supplemental oxygen with 2 liters on nasal cannula, broad spectrum antibiotics (piperacillin/tazobactam and vancomycin), antipyretics, and multiple doses of IV lorazepam with the aim of treating his tachycardia with a mixed picture of sepsis, dehydration, and methamphetamine intoxication.

Point-of-care ultrasound (POCUS) of the inferior vena cava (IVC), heart, and lungs was obtained. The IVC diameter measured 1.85 cm (max) in inspiration and 1.71 cm (min) in expiration (Images 1-2, Video 1). Cardiac views demonstrated gross biatrial and biventricular dilation on visual inspection with a severely reduced left ventricular ejection fraction (LVEF) and a small pericardial effusion (Images 3-5, Videos 2-3). Lung ultrasound demonstrated bilateral lung sliding anteriorly and B-lines in bilateral inferolateral lung fields (Images 6-7, Videos 4-5). With the information obtained via US, the decision was made to consult cardiology and to restrict IVF administration by giving the patient 1 liter of normal saline over the course of two hours instead of a 2-liter bolus.

Electrocardiogram (ECG) revealed sinus tachycardia with a normal axis and without ST elevations, PR depressions, or significant T-wave inversions (Image 8). Chest X-ray demonstrated a right lower lobe consolidation with evidence of central pulmonary venous congestion and cephalization (Image 9). Significant laboratory findings included a WBC count of 21,000 with a neutrophilic predominance of 81%, lactic acid of 1.8 mmol/L, Pro-BNP of 1,741 pg/ml, and Troponin I < 0.04 ng/ml. The rest of the basic blood works including renal function and electrolytes were within normal limits for our institution. Computed tomography (CT) pulmonary angiogram was performed and demonstrated a right lower lobe consolidation and no evidence of pulmonary embolism. Also evident on CT were atrial and ventricular enlargement, central pulmonary venous congestion, and signs of early pulmonary edema (Images 10-11).

The patient was admitted to the ICU and was continued on broad-spectrum antibiotics and a normal saline infusion of 75 ml/hr. On day two, all IV fluids were discontinued and diuresis was employed for decompensated heart failure with developing hypoxia. The patient grew 2/2 positive blood cultures for* Streptococcus pneumoniae* and was found to be Influenza B positive. Comprehensive 2D Echocardiogram revealed dilated cardiomyopathy with LVEF of 16%, restrictive left ventricular filling pattern, elevated filling pressures with E/e'=30, moderate/severe mitral regurgitation, and severe tricuspid regurgitation.

The patient improved during hospitalization and was discharged on day 9 with a diagnosis of severe sepsis, right lower lobe pneumonia,* Streptococcus pneumoniae* bacteremia, Influenza B, and Methamphetamine-Associated Cardiomyopathy.

He was discharged on antibiotic therapy and a heart failure regimen consisting of lisinopril and metoprolol.

His cardiac dysfunction improved with drug abstinence and medical therapy. A four-month echocardiogram demonstrated resolution of dilated atrial and ventricular chambers, with improvement of LVEF to 45% and improvement in valvular dysfunction to only trace mitral and tricuspid regurgitation.

## 3. Discussion

Point-of-care ultrasound (POCUS) has brought significant advances in the assessment of critically ill patients and continues to gain support and popularity across specialties [1–4]. The integration of ultrasound in the management of critically ill patients with sepsis and undifferentiated hypotension has been demonstrated to increase physician diagnostic accuracy, influence physician decision making, and improve patient outcomes.[5–8] Appropriate assessment in determining who needs fluid resuscitation is an important skill emergency physicians must own. There is a growing body of literature that demonstrates that excessive fluid resuscitation can lead to worsening respiratory function, renal injury, and overall increase in morbidity and mortality.[9–13] In light of the uncertainty of evidence to support point-of-care ultrasound in predicting fluid responsiveness, we highlight how ultrasound played a pivotal role in how we guided fluid resuscitation in this case.

Despite this patient's clinical presentation and our initial impression, the ultrasound findings discovered were not suggestive of a patient who would benefit from large fluid resuscitation. Furthermore, there were sonographic findings suggestive of decompensated heart failure. Cardiac evaluation demonstrated an unsuspected dilated cardiomyopathy with a severe globally depressed contractility.

The reliability IVC sonography to assess right atrial pressure has been well established and is encouraged in the latest update from The American Society of Echocardiography and European Association of Cardiovascular Imaging to estimate right atrial pressure.[14] However, the reliability of IVC assessment to predict fluid responsiveness has been brought into question and is far from perfect. Literature shows conflicting evidence with heterogeneity and variability in methods and conclusions.[15] Many factors affect and add variability to IVC CI including ventilatory status, age, body habitus, and the presence of concomitant disease processes.[16] A systematic review and meta-analysis from 2016 determined that when using a mean IVC CI of 40-50% in spontaneously breathing patients, it performed moderately well with an AUROC of 0.76.[15] This patient's IVC CI was 7.5%, which was far from the popularly accepted 40-50% cut-off observed in literature.[15] Other emerging methods to assess fluid responsiveness would have likely been useful in this case but were not performed. Dynamic monitoring with ultrasound can aid in evaluating response to fluid challenges via assessing changes in IVC, progression or resolution of B-lines, and cardiac output measurements[17] Additional sonographic evaluations during resuscitation to evaluate progression in B-lines and IVC changes would have likely been helpful in this case with regard to his response to current management. A passive leg raise can provide a “reversible” fluid bolus which demonstrated high reliability in predicting fluid responsiveness with cardiac output measurements without the risk of irreversible IV fluid boluses.[18] Our patient's respiratory distress would not make him tolerable to lying flat which is required during this technique and is one of its imitations. Subaortic and carotid Velocity Time Integral (VTI) with a passive leg raise (PLR) has shown promising reliability; however measurements are more technically challenging and are more time-consuming to perform.[17]

Aside from fluid responsiveness, there have been multiple prospective studies investigating the accuracy of IVC assessment in diagnosing heart failure. A summary paper on the accuracy of IVC measurements in detecting congestive heart failure calculated 84-96% specificity when using IVC CI cut-off of <15% to identify congestive heart failure.[19] Our patient's IVC CI cut-off as 7.5% met the former mentioned criteria, supporting the diagnosis of decompensated heart failure.

Lung ultrasound (LUS) was the third component evaluated. US is an expanding field allowing rapid and reliable bedside assessment for a variety of lung pathologies.[20] Using LUS to detect pulmonary edema has shown diagnostic utility and may have higher specificity than chest X-ray.[21–23] A recent meta-analysis determined lung ultrasound as a viable modality for diagnosing decompensated heart failure in undifferentiated emergency department patients with a sensitivity of 82.5 % and specificity of 83.6%.[23] The International Expert Consensus for Lung Ultrasound defines a positive study for interstitial syndrome is accepted as the presence of 3 or more B-lines per lung field, in 2 or more fields bilaterally.[21] B-lines were present in the right and left inferolateral lung fields but absent on the anterior chest fields that we imaged. Our lung ultrasound findings technically do not meet the definition of interstitial syndrome, since we only had 2 out of 4 positive lung fields; however, we believe that, given the time to examine more lung fields, we would have likely discovered more regions of B-lines. It is important to note the operator time required to perform these studies is a fundamental limitation in performing diagnostic ultrasound and is not always feasible. The addition of further diagnostic information increases the accuracy of detecting pulmonary edema and differentiating between other etiologies that can cause sonographic interstitial syndrome including pneumonia and ARDS.[22, 23] An observational study showed that the combination of B-lines in the lateral lung fields in combination with an elevated Pro-BNP carried a positive LR ratio >8 for the presence of congestive heart failure, which was consistent in this case.[24]

The information provided from our cardiac, lung, and IVC ultrasound changed our initial impression and management in this case. This patient's methamphetamine abuse complicated his clinical course of severe sepsis by pushing him into decompensated heart failure from underlying Methamphetamine-Associated Cardiomyopathy (MAC). Utilizing solely the history, physical, and standard ED evaluation tools (CXR, CT, labs, etc.) would have likely delayed identification of his congestive heart failure component and allowed potentially deleterious fluid resuscitation. With a total of 102,961 ED visits involving methamphetamine usage in 2011 and a significant rise in prevalence of methamphetamine usage in the United states, ED physicians should be alert to this condition.[25] Lack of awareness may lead to misdiagnosis and worsened outcomes in these at-risk patients presenting to the emergency department.

Methamphetamine abuse has been linked to a variety of cardiovascular pathologies including hypertension, tachycardia, cardiac arrhythmias, acute coronary syndrome, pulmonary hypertension, aortic dissection, sudden cardiac death, and cardiomyopathy.[26] Desired euphoric, hallucinogenic, anorexigenic, and stimulant-like effects make them a popular drug of abuse.[26] The association between amphetamine abuse and cardiomyopathy was first described in 1979 by Smith et al.[27] Since then, there has been various publications on Methamphetamine-Associated Cardiomyopathy (MAC), also described as Amphetamine-Associated Cardiomyopathy (AAC), but they are mostly limited to animal studies, case reports, case series, and case-control studies.[28] Methamphetamine users have a 3.7 times greater risk of developing cardiomyopathy and greater than 5% of patients hospitalized for heart failure report stimulant abuse.[29] Nonischemic dilated cardiomyopathy is by far the most commonly reported variant associated with methamphetamine abuse; however other forms have been reported including stress cardiomyopathy (Takotsubo) and hypertrophic cardiomyopathy.[27] The risk in developing specific cardiomyopathies may be influenced by genetic predisposition.[27] The majority of patients diagnosed are young males who present late with severe dysfunction.[28]

The underlying process leading to development of MAC has been postulated with various mechanisms and likely has multiple contributing factors. Methamphetamines are sympathomimetic amines that increase levels of intrasynaptic monoamines (epinephrine, norepinephrine, dopamine, and serotonin) via multiple mechanisms.[29] Cardiac damage by catecholamine excess inducing vasospasm and ischemia, direct cardiac myocyte toxicity, impaired cellular metabolism, elevated reactive oxygen species (ROS), and mitochondrial injury have all been proposed mechanism.[29] Over time cardiac myocytic degeneration and contract band necrosis lead to decreased systolic function and resultant dilated cardiomyopathy.[30] It has been shown that the cardiac remodeling associated with methamphetamine abuse can be reversed with B-blockers and ACE-inhibitor use after methamphetamine use cessation; however the rate and time of recovery are variable.[29] Cardiac magnetic resonance imaging by late gadolinium enhancement can evaluate the extent of contract band necrosis and fibrosis which likely determines the heart's ability to recover when the drug of abuse is stopped.[31]

We acknowledge that this case was complex with multiple factors likely involved leading to his acute presentation in the emergency department. A presentation of endocarditis was considered; however it was felt to be less likely given the patient denied intravenous drug use and there were no large vegetations seen on cardiac ultrasound. We suspect sepsis played a contributing role in this patient's acute presentation of decompensated cardiomyopathy; however, there are certain findings in this case that support underlying Methamphetamine-Associated Cardiomyopathy over a stand-alone diagnosis of Sepsis-Induced Cardiomyopathy. Along with the patient's history of heavy methamphetamine use, his echocardiogram displayed findings commonly seen in methamphetamine induced cardiomyopathy with chronicity of his condition evident by a restrictive left ventricular filling pattern that is not expected to be seen in an acute process such as Sepsis-Induced Cardiomyopathy.[28, 29, 32] At 4 months, the patient still displayed a reduced left ventricular ejection fraction (LVEF) of 45%. This is also not expected in Sepsis-Induced Cardiomyopathy, in which LVEF is, by definition, required to return to baseline cardiac function within 2 weeks.[33] Cardiology suspected the diagnosis of MAC and attempted to obtain a late gadolinium enhanced cardiac MRI during his hospitalization which would have supported the diagnosis. The patient unfortunately did not tolerate this test and it was never completed.

This case highlights how POCUS identified and changed management of an infrequent ED presentation of decompensated heart failure in a young male with previously undiagnosed Methamphetamine-Associated Cardiomyopathy complicated with severe sepsis. Methamphetamine-Associated Cardiomyopathy (MAC) is an infrequently described complication of methamphetamine abuse, which has a significant impact on morbidity amongst users. With the rise in methamphetamine abuse in the United States, emergency physicians should be alert to this condition. It is important that early recognition be made in the emergency department so proper education and rehabilitation, with a goal of abstinence from amphetamine use, may allow patients to potentially regain normal cardiac function.[9, 25] Additionally, early detection is essential amongst patients who present critically ill since recognition may impact the resuscitation strategy.

Assessing volume status and predicting fluid responsiveness in critically ill patients are an important clinical question that has been a highly discussed topic amongst the emergency medicine and the critical care community. The ideal modality should be fast, reliable, noninvasive, inexpensive, easily obtainable at bedside, and reproducible amongst a wide variety of providers of various levels' training. Unfortunately, there is no ideal modality and each technique has its limitations. Many emerging and promising methods are being explored including passive leg raise testing techniques, changes in end tidal CO2 measurements, and noninvasive cardiac output monitoring devices, which are areas of future interest.[17] Despite the ambiguity in literature regarding fluid responsiveness, we highlight how ultrasound can be a valuable tool in assessment of critical patients and guide resuscitation in a complex clinical scenario in which multiple present disease processes are treated contrastingly. Resuscitation strategies often well precede results from diagnostic information such as blood work and diagnostic radiology. POCUS provides prompt and invaluable information that can be obtained at bedside during the initial evaluation of a critical patient, well before other formal diagnostic information has returned. This can make bedside ultrasound an important tool in guiding an emergency physician's initial resuscitation strategy. Without the information provided by POCUS, the patient's underlying cardiomyopathy would have likely had a significant delay in diagnosis or even gone unrecognized in the emergency department. Administration of excessive fluid in the emergency department and during hospitalization would have likely lead to worsening pulmonary edema. The impact of this could have increased his hospital length of stay and may have even necessitated the need for ventilatory support. Early identification of his underlying cardiomyopathy by using POCUS in the ED appropriated restrictive fluid resuscitation prompted early cardiology consultation and likely improved this patient's overall outcome. Even though no randomized controlled trials exist, this case also supports that ultrasound may be a more sensitive tool to detect early pulmonary edema when compared with chest X-ray. The initial chest X-ray had only subtle findings when compared with the lung ultrasound, which had very obvious findings of interstitial edema. This is an area for future research considerations.

## Data Availability

The authors declare that data supporting the findings of this study are available within the article and its supplementary information files.

## Supplementary Materials

Supplementary Materials Image 1: ultrasound of long axis view of IVC in inspiration: maximal internal diameter of 1.71 cm. Image 2: ultrasound of long axis view of IVC in expiration: maximal internal diameter of 1.85 cm. Video 1: ultrasound long axis view of IVC: dilated with minimal respiratory variation. Image 3: ultrasound of parasternal long axis (PLAX) view of heart: biventricular dilation. Image 4: ultrasound of parasternal short axis (PSAX) view of heart: biventricular dilation and small pericardial effusion. Image 5: ultrasound of apical four chamber (A4C) view of heart view: biatrial and biventricular dilation. Video 2: ultrasound of apical four-chamber (A4C) view of heart view: biatrial and biventricular dilation with severely depressed systolic function. Video 3: ultrasound of parasternal short axis (PSAX) view of heart: biventricular dilation and small pericardial effusion. Image 6: lung ultrasound of right inferolateral lung field: prominent B-lines. Image 7: lung ultrasound of left inferolateral lung field: prominent B-lines. Video 4: lung ultrasound of right inferolateral lung field: prominent B-lines. Video 5: lung ultrasound of left inferolateral lung field: prominent B-lines. Image 8: ECG: sinus tachycardia. No evidence of pericarditis. Image 9: chest X-ray: right lower lobe consolidation with evidence of central pulmonary venous congestion and cephalization. Image 10: computed tomography (CT) pulmonary angiogram (angiography window): right lower lobe consolidation, and no evidence of pulmonary embolism. Image 11: computed tomography (CT) pulmonary angiogram (lung window): right lower lobe consolidation, small pericardial effusion, and atrial and ventricular enlargement. Central pulmonary venous congestion and signs of early pulmonary edema also evident.
