# Supplementary figures and images for: Point-of-Care Ultrasound Identifies Decompensated Heart Failure in a Young Male with Methamphetamine-Associated Cardiomyopathy Presenting in Severe Sepsis to the Emergency Department
Source: Case Rep Emerg Med. 2018 Oct 9;2018:2859676. doi: 10.1155/2018/2859676 (PMC6198549; doi:10.1155/2018/2859676)

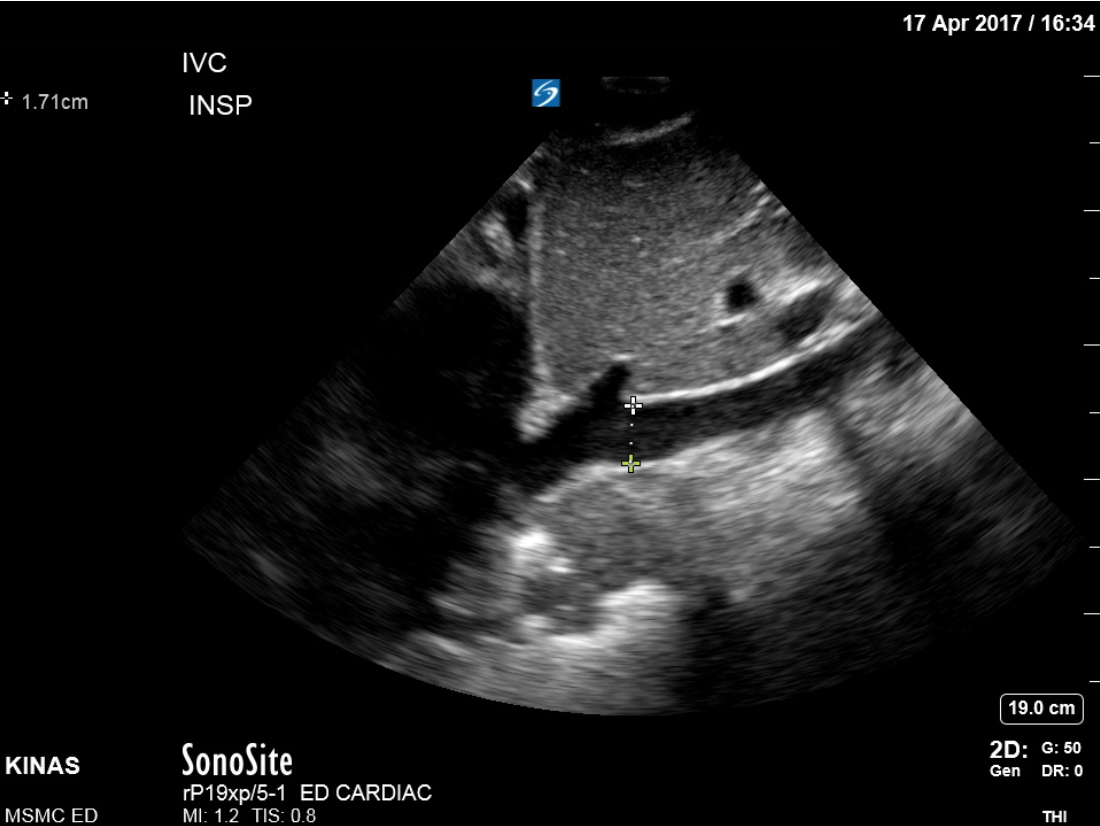

Supplement: Supplementary Materials — Image 1: ultrasound of long axis view of IVC in inspiration: maximal internal diameter of 1.71 cm. Image 2: ultrasound of long axis view of IVC in expiration: maximal internal diameter of 1.85 cm. Video 1: ultrasound long axis view of IVC: dilated with minimal respiratory variation. Image 3: ultrasound of parasternal long axis (PLAX) view of heart: biventricular dilation. Image 4: ultrasound of parasternal short axis (PSAX) view of heart: biventricular dilation and small pericardial effusion. Image 5: ultrasound of apical four chamber (A4C) view of heart view: biatrial and biventricular dilation. Video 2: ultrasound of apical four-chamber (A4C) view of heart view: biatrial and biventricular dilation with severely depressed systolic function. Video 3: ultrasound of parasternal short axis (PSAX) view of heart: biventricular dilation and small pericardial effusion. Image 6: lung ultrasound of right inferolateral lung field: prominent B-lines. Image 7: lung ultrasound of left inferolateral lung field: prominent B-lines. Video 4: lung ultrasound of right inferolateral lung field: prominent B-lines. Video 5: lung ultrasound of left inferolateral lung field: prominent B-lines. Image 8: ECG: sinus tachycardia. No evidence of pericarditis. Image 9: chest X-ray: right lower lobe consolidation with evidence of central pulmonary venous congestion and cephalization. Image 10: computed tomography (CT) pulmonary angiogram (angiography window): right lower lobe consolidation, and no evidence of pulmonary embolism. Image 11: computed tomography (CT) pulmonary angiogram (lung window): right lower lobe consolidation, small pericardial effusion, and atrial and ventricular enlargement. Central pulmonary venous congestion and signs of early pulmonary edema also evident. [file 2859676.f1.zip › 2859676.f1/Image 1.jpg]

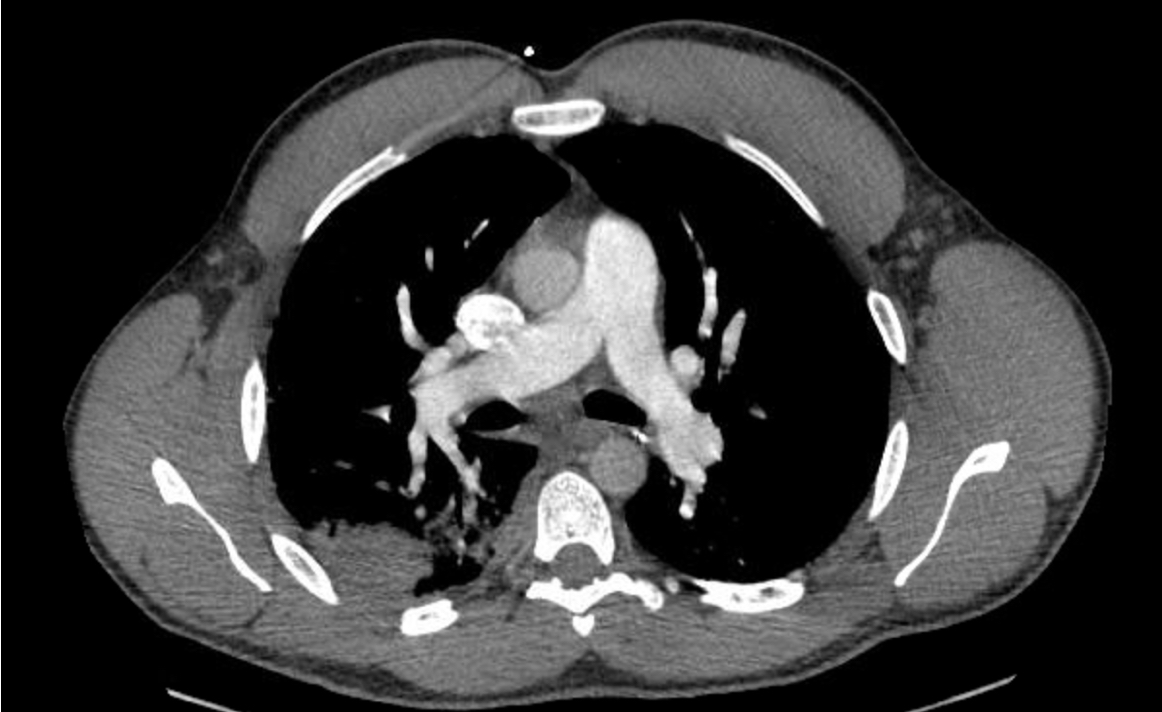

Supplement: Supplementary Materials — Image 1: ultrasound of long axis view of IVC in inspiration: maximal internal diameter of 1.71 cm. Image 2: ultrasound of long axis view of IVC in expiration: maximal internal diameter of 1.85 cm. Video 1: ultrasound long axis view of IVC: dilated with minimal respiratory variation. Image 3: ultrasound of parasternal long axis (PLAX) view of heart: biventricular dilation. Image 4: ultrasound of parasternal short axis (PSAX) view of heart: biventricular dilation and small pericardial effusion. Image 5: ultrasound of apical four chamber (A4C) view of heart view: biatrial and biventricular dilation. Video 2: ultrasound of apical four-chamber (A4C) view of heart view: biatrial and biventricular dilation with severely depressed systolic function. Video 3: ultrasound of parasternal short axis (PSAX) view of heart: biventricular dilation and small pericardial effusion. Image 6: lung ultrasound of right inferolateral lung field: prominent B-lines. Image 7: lung ultrasound of left inferolateral lung field: prominent B-lines. Video 4: lung ultrasound of right inferolateral lung field: prominent B-lines. Video 5: lung ultrasound of left inferolateral lung field: prominent B-lines. Image 8: ECG: sinus tachycardia. No evidence of pericarditis. Image 9: chest X-ray: right lower lobe consolidation with evidence of central pulmonary venous congestion and cephalization. Image 10: computed tomography (CT) pulmonary angiogram (angiography window): right lower lobe consolidation, and no evidence of pulmonary embolism. Image 11: computed tomography (CT) pulmonary angiogram (lung window): right lower lobe consolidation, small pericardial effusion, and atrial and ventricular enlargement. Central pulmonary venous congestion and signs of early pulmonary edema also evident. [file 2859676.f1.zip › 2859676.f1/Image 10.jpg]

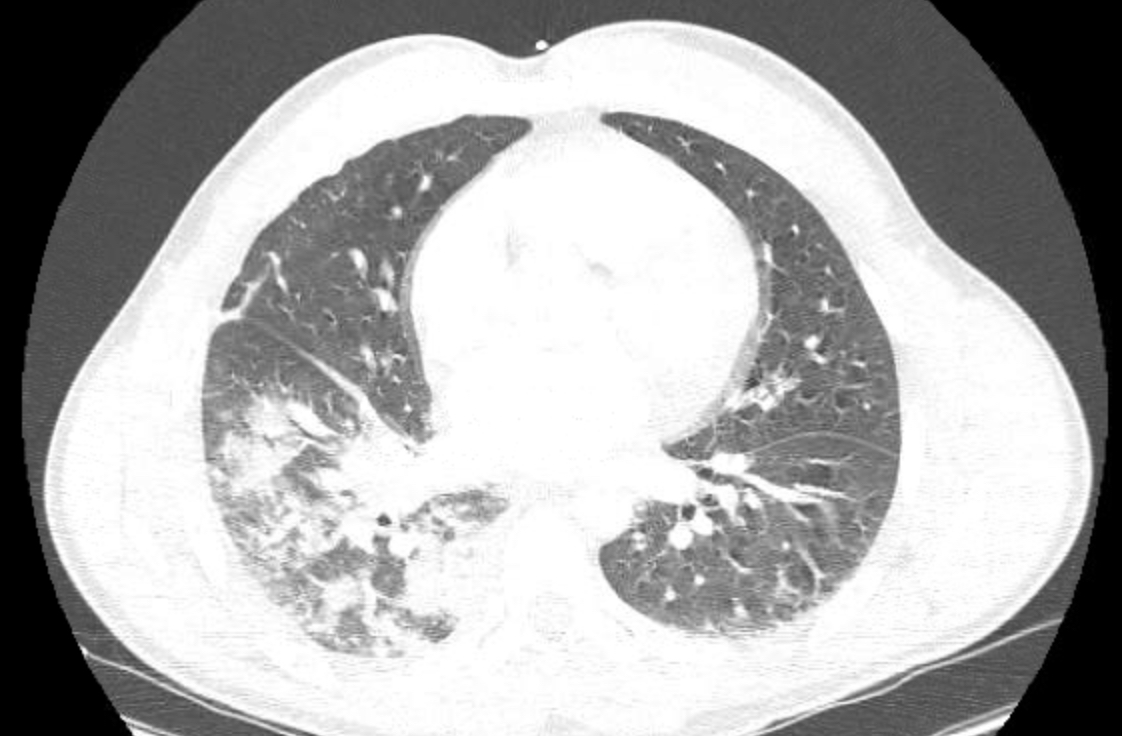

Supplement: Supplementary Materials — Image 1: ultrasound of long axis view of IVC in inspiration: maximal internal diameter of 1.71 cm. Image 2: ultrasound of long axis view of IVC in expiration: maximal internal diameter of 1.85 cm. Video 1: ultrasound long axis view of IVC: dilated with minimal respiratory variation. Image 3: ultrasound of parasternal long axis (PLAX) view of heart: biventricular dilation. Image 4: ultrasound of parasternal short axis (PSAX) view of heart: biventricular dilation and small pericardial effusion. Image 5: ultrasound of apical four chamber (A4C) view of heart view: biatrial and biventricular dilation. Video 2: ultrasound of apical four-chamber (A4C) view of heart view: biatrial and biventricular dilation with severely depressed systolic function. Video 3: ultrasound of parasternal short axis (PSAX) view of heart: biventricular dilation and small pericardial effusion. Image 6: lung ultrasound of right inferolateral lung field: prominent B-lines. Image 7: lung ultrasound of left inferolateral lung field: prominent B-lines. Video 4: lung ultrasound of right inferolateral lung field: prominent B-lines. Video 5: lung ultrasound of left inferolateral lung field: prominent B-lines. Image 8: ECG: sinus tachycardia. No evidence of pericarditis. Image 9: chest X-ray: right lower lobe consolidation with evidence of central pulmonary venous congestion and cephalization. Image 10: computed tomography (CT) pulmonary angiogram (angiography window): right lower lobe consolidation, and no evidence of pulmonary embolism. Image 11: computed tomography (CT) pulmonary angiogram (lung window): right lower lobe consolidation, small pericardial effusion, and atrial and ventricular enlargement. Central pulmonary venous congestion and signs of early pulmonary edema also evident. [file 2859676.f1.zip › 2859676.f1/Image 11.jpg]

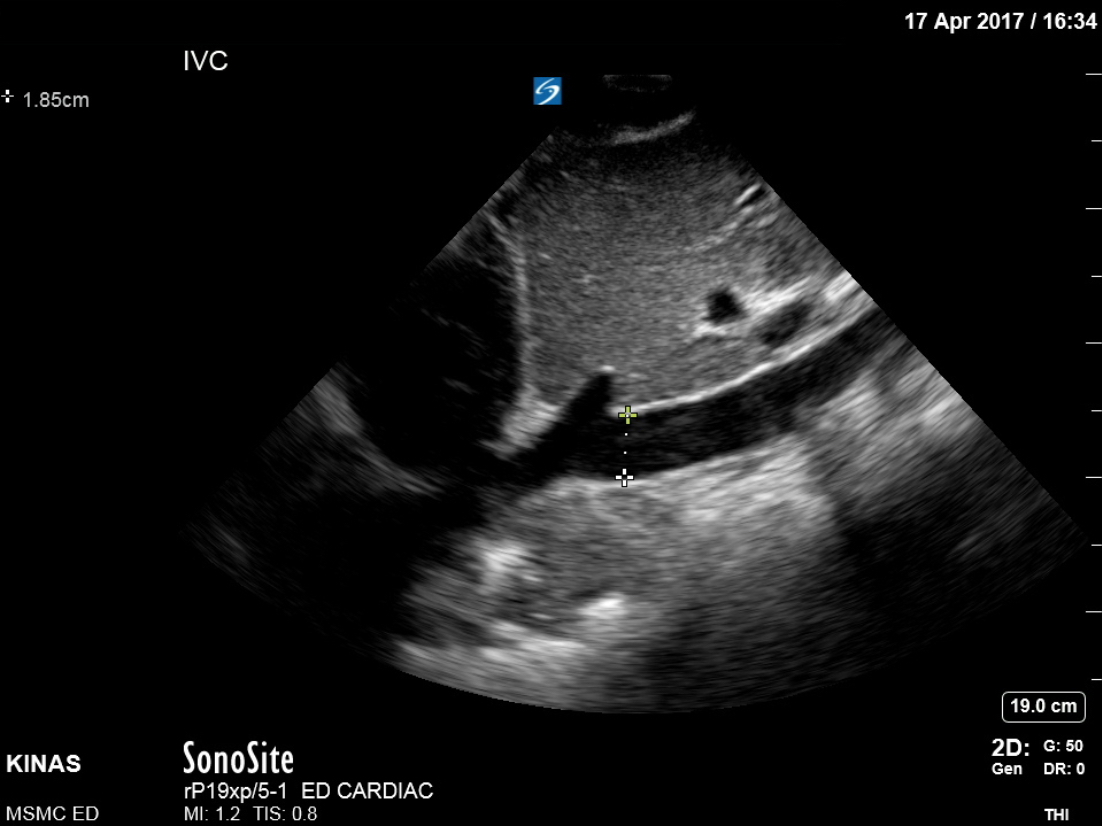

Supplement: Supplementary Materials — Image 1: ultrasound of long axis view of IVC in inspiration: maximal internal diameter of 1.71 cm. Image 2: ultrasound of long axis view of IVC in expiration: maximal internal diameter of 1.85 cm. Video 1: ultrasound long axis view of IVC: dilated with minimal respiratory variation. Image 3: ultrasound of parasternal long axis (PLAX) view of heart: biventricular dilation. Image 4: ultrasound of parasternal short axis (PSAX) view of heart: biventricular dilation and small pericardial effusion. Image 5: ultrasound of apical four chamber (A4C) view of heart view: biatrial and biventricular dilation. Video 2: ultrasound of apical four-chamber (A4C) view of heart view: biatrial and biventricular dilation with severely depressed systolic function. Video 3: ultrasound of parasternal short axis (PSAX) view of heart: biventricular dilation and small pericardial effusion. Image 6: lung ultrasound of right inferolateral lung field: prominent B-lines. Image 7: lung ultrasound of left inferolateral lung field: prominent B-lines. Video 4: lung ultrasound of right inferolateral lung field: prominent B-lines. Video 5: lung ultrasound of left inferolateral lung field: prominent B-lines. Image 8: ECG: sinus tachycardia. No evidence of pericarditis. Image 9: chest X-ray: right lower lobe consolidation with evidence of central pulmonary venous congestion and cephalization. Image 10: computed tomography (CT) pulmonary angiogram (angiography window): right lower lobe consolidation, and no evidence of pulmonary embolism. Image 11: computed tomography (CT) pulmonary angiogram (lung window): right lower lobe consolidation, small pericardial effusion, and atrial and ventricular enlargement. Central pulmonary venous congestion and signs of early pulmonary edema also evident. [file 2859676.f1.zip › 2859676.f1/Image 2.jpg]

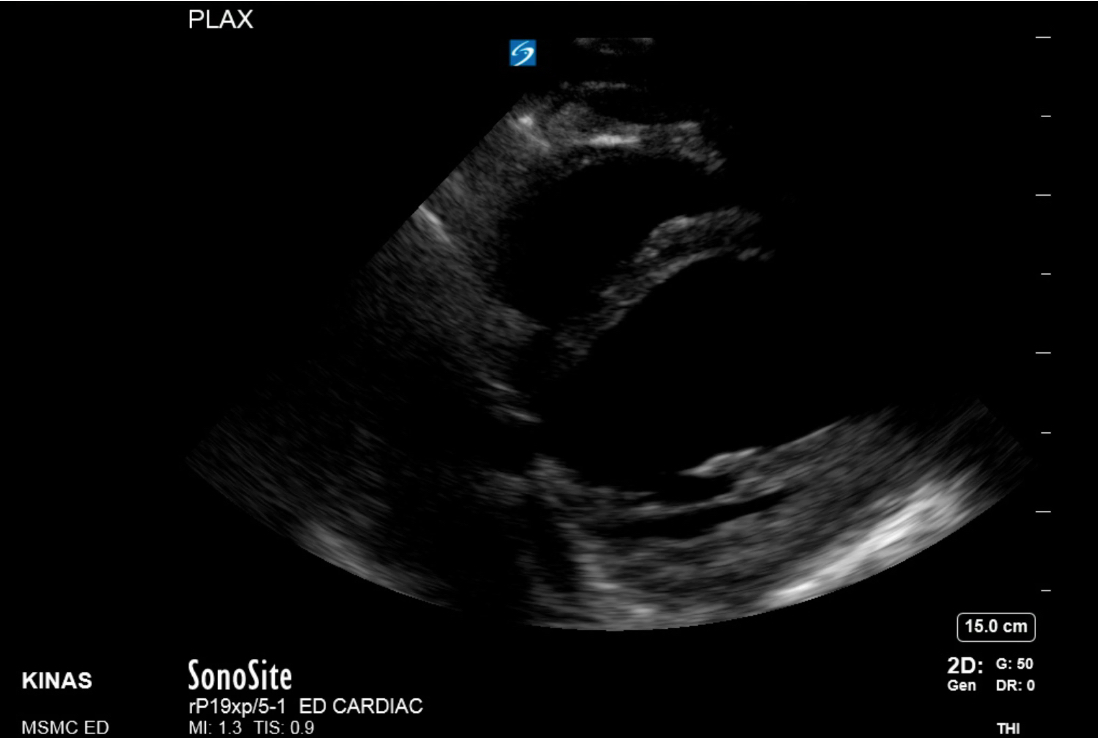

Supplement: Supplementary Materials — Image 1: ultrasound of long axis view of IVC in inspiration: maximal internal diameter of 1.71 cm. Image 2: ultrasound of long axis view of IVC in expiration: maximal internal diameter of 1.85 cm. Video 1: ultrasound long axis view of IVC: dilated with minimal respiratory variation. Image 3: ultrasound of parasternal long axis (PLAX) view of heart: biventricular dilation. Image 4: ultrasound of parasternal short axis (PSAX) view of heart: biventricular dilation and small pericardial effusion. Image 5: ultrasound of apical four chamber (A4C) view of heart view: biatrial and biventricular dilation. Video 2: ultrasound of apical four-chamber (A4C) view of heart view: biatrial and biventricular dilation with severely depressed systolic function. Video 3: ultrasound of parasternal short axis (PSAX) view of heart: biventricular dilation and small pericardial effusion. Image 6: lung ultrasound of right inferolateral lung field: prominent B-lines. Image 7: lung ultrasound of left inferolateral lung field: prominent B-lines. Video 4: lung ultrasound of right inferolateral lung field: prominent B-lines. Video 5: lung ultrasound of left inferolateral lung field: prominent B-lines. Image 8: ECG: sinus tachycardia. No evidence of pericarditis. Image 9: chest X-ray: right lower lobe consolidation with evidence of central pulmonary venous congestion and cephalization. Image 10: computed tomography (CT) pulmonary angiogram (angiography window): right lower lobe consolidation, and no evidence of pulmonary embolism. Image 11: computed tomography (CT) pulmonary angiogram (lung window): right lower lobe consolidation, small pericardial effusion, and atrial and ventricular enlargement. Central pulmonary venous congestion and signs of early pulmonary edema also evident. [file 2859676.f1.zip › 2859676.f1/Image 3.jpg]

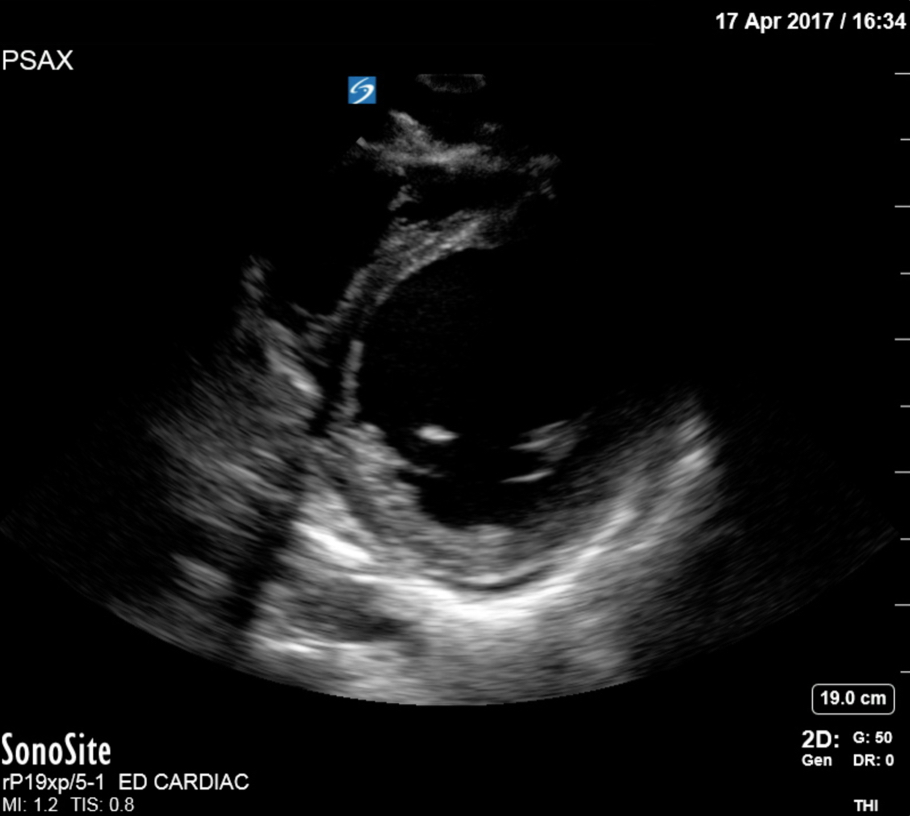

Supplement: Supplementary Materials — Image 1: ultrasound of long axis view of IVC in inspiration: maximal internal diameter of 1.71 cm. Image 2: ultrasound of long axis view of IVC in expiration: maximal internal diameter of 1.85 cm. Video 1: ultrasound long axis view of IVC: dilated with minimal respiratory variation. Image 3: ultrasound of parasternal long axis (PLAX) view of heart: biventricular dilation. Image 4: ultrasound of parasternal short axis (PSAX) view of heart: biventricular dilation and small pericardial effusion. Image 5: ultrasound of apical four chamber (A4C) view of heart view: biatrial and biventricular dilation. Video 2: ultrasound of apical four-chamber (A4C) view of heart view: biatrial and biventricular dilation with severely depressed systolic function. Video 3: ultrasound of parasternal short axis (PSAX) view of heart: biventricular dilation and small pericardial effusion. Image 6: lung ultrasound of right inferolateral lung field: prominent B-lines. Image 7: lung ultrasound of left inferolateral lung field: prominent B-lines. Video 4: lung ultrasound of right inferolateral lung field: prominent B-lines. Video 5: lung ultrasound of left inferolateral lung field: prominent B-lines. Image 8: ECG: sinus tachycardia. No evidence of pericarditis. Image 9: chest X-ray: right lower lobe consolidation with evidence of central pulmonary venous congestion and cephalization. Image 10: computed tomography (CT) pulmonary angiogram (angiography window): right lower lobe consolidation, and no evidence of pulmonary embolism. Image 11: computed tomography (CT) pulmonary angiogram (lung window): right lower lobe consolidation, small pericardial effusion, and atrial and ventricular enlargement. Central pulmonary venous congestion and signs of early pulmonary edema also evident. [file 2859676.f1.zip › 2859676.f1/Image 4.jpg]

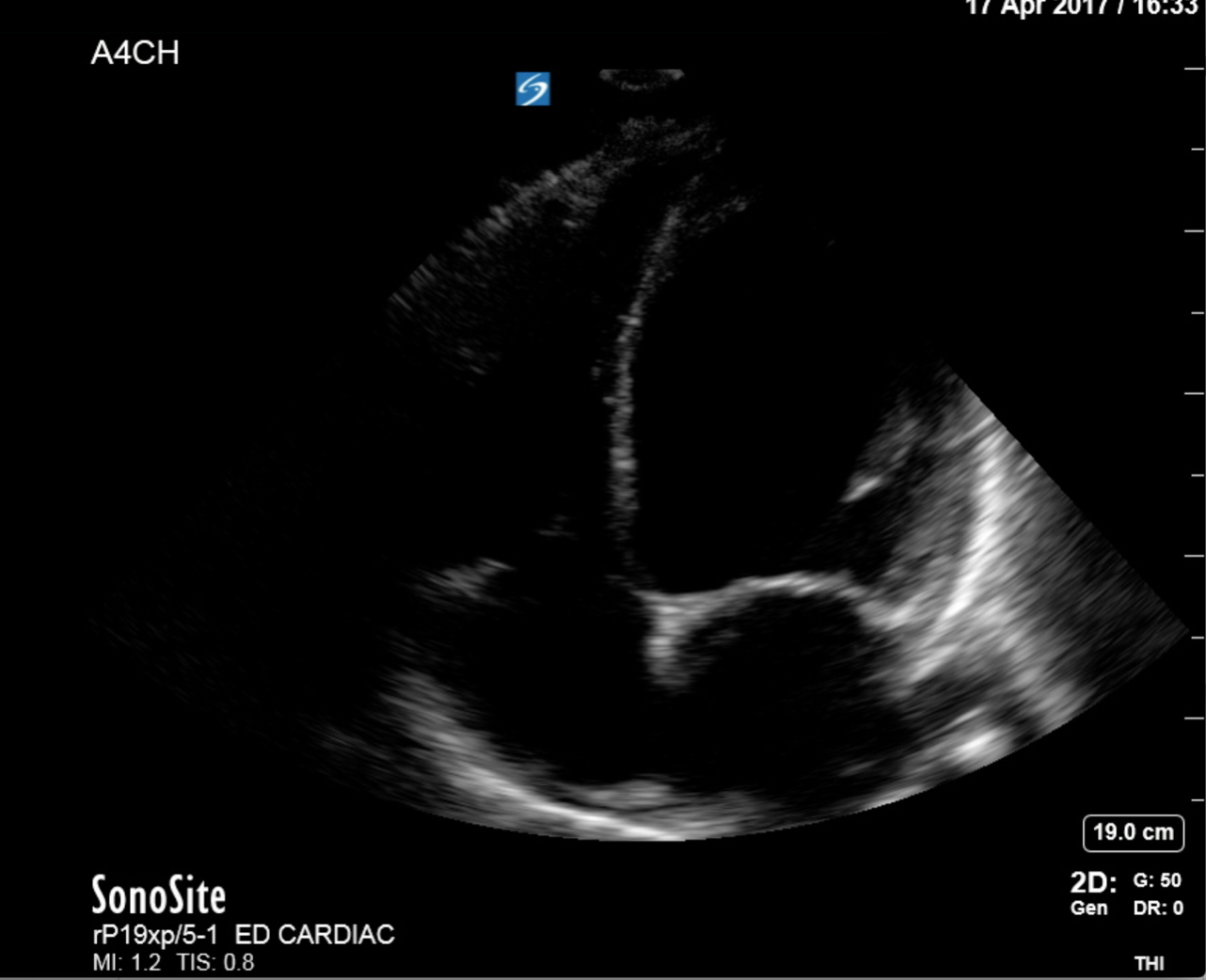

Supplement: Supplementary Materials — Image 1: ultrasound of long axis view of IVC in inspiration: maximal internal diameter of 1.71 cm. Image 2: ultrasound of long axis view of IVC in expiration: maximal internal diameter of 1.85 cm. Video 1: ultrasound long axis view of IVC: dilated with minimal respiratory variation. Image 3: ultrasound of parasternal long axis (PLAX) view of heart: biventricular dilation. Image 4: ultrasound of parasternal short axis (PSAX) view of heart: biventricular dilation and small pericardial effusion. Image 5: ultrasound of apical four chamber (A4C) view of heart view: biatrial and biventricular dilation. Video 2: ultrasound of apical four-chamber (A4C) view of heart view: biatrial and biventricular dilation with severely depressed systolic function. Video 3: ultrasound of parasternal short axis (PSAX) view of heart: biventricular dilation and small pericardial effusion. Image 6: lung ultrasound of right inferolateral lung field: prominent B-lines. Image 7: lung ultrasound of left inferolateral lung field: prominent B-lines. Video 4: lung ultrasound of right inferolateral lung field: prominent B-lines. Video 5: lung ultrasound of left inferolateral lung field: prominent B-lines. Image 8: ECG: sinus tachycardia. No evidence of pericarditis. Image 9: chest X-ray: right lower lobe consolidation with evidence of central pulmonary venous congestion and cephalization. Image 10: computed tomography (CT) pulmonary angiogram (angiography window): right lower lobe consolidation, and no evidence of pulmonary embolism. Image 11: computed tomography (CT) pulmonary angiogram (lung window): right lower lobe consolidation, small pericardial effusion, and atrial and ventricular enlargement. Central pulmonary venous congestion and signs of early pulmonary edema also evident. [file 2859676.f1.zip › 2859676.f1/Image 5.jpg]

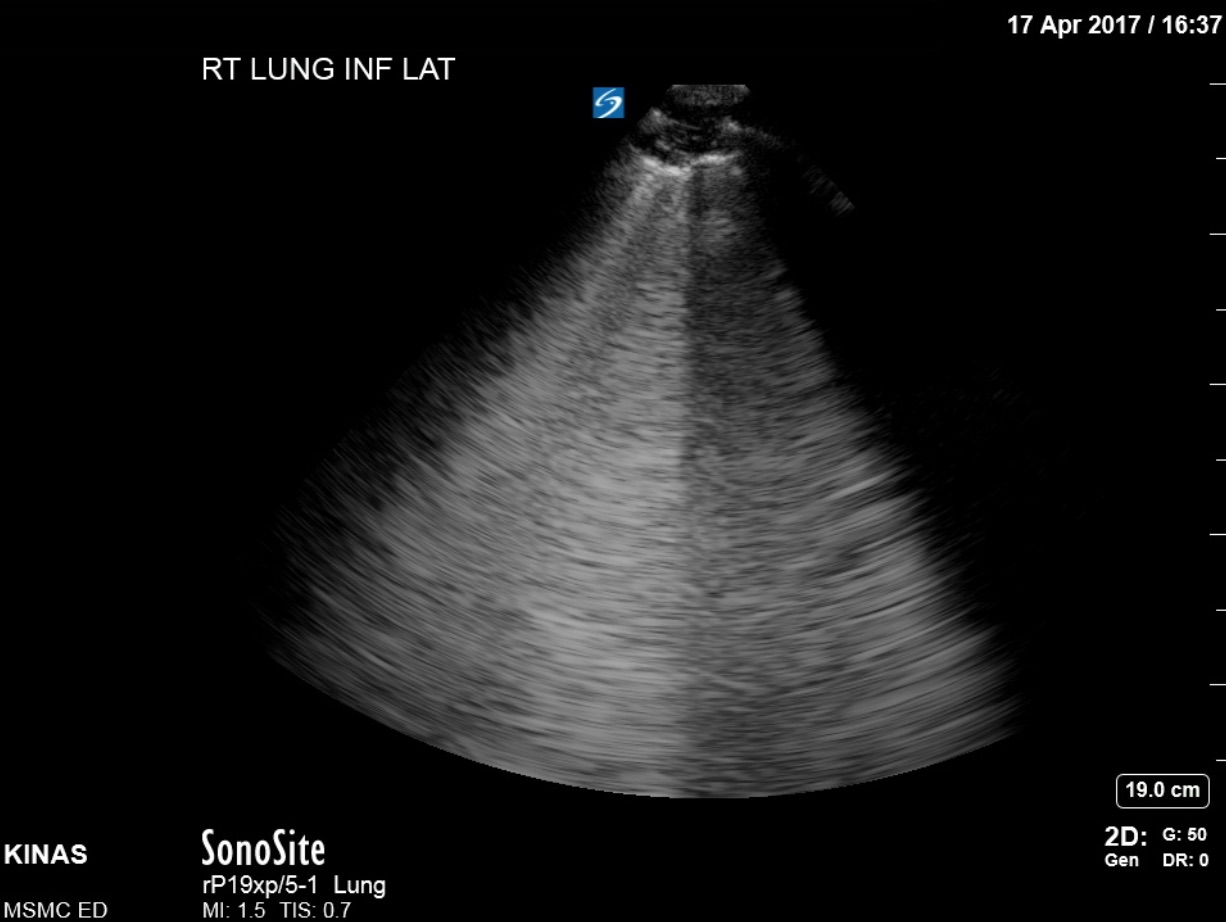

Supplement: Supplementary Materials — Image 1: ultrasound of long axis view of IVC in inspiration: maximal internal diameter of 1.71 cm. Image 2: ultrasound of long axis view of IVC in expiration: maximal internal diameter of 1.85 cm. Video 1: ultrasound long axis view of IVC: dilated with minimal respiratory variation. Image 3: ultrasound of parasternal long axis (PLAX) view of heart: biventricular dilation. Image 4: ultrasound of parasternal short axis (PSAX) view of heart: biventricular dilation and small pericardial effusion. Image 5: ultrasound of apical four chamber (A4C) view of heart view: biatrial and biventricular dilation. Video 2: ultrasound of apical four-chamber (A4C) view of heart view: biatrial and biventricular dilation with severely depressed systolic function. Video 3: ultrasound of parasternal short axis (PSAX) view of heart: biventricular dilation and small pericardial effusion. Image 6: lung ultrasound of right inferolateral lung field: prominent B-lines. Image 7: lung ultrasound of left inferolateral lung field: prominent B-lines. Video 4: lung ultrasound of right inferolateral lung field: prominent B-lines. Video 5: lung ultrasound of left inferolateral lung field: prominent B-lines. Image 8: ECG: sinus tachycardia. No evidence of pericarditis. Image 9: chest X-ray: right lower lobe consolidation with evidence of central pulmonary venous congestion and cephalization. Image 10: computed tomography (CT) pulmonary angiogram (angiography window): right lower lobe consolidation, and no evidence of pulmonary embolism. Image 11: computed tomography (CT) pulmonary angiogram (lung window): right lower lobe consolidation, small pericardial effusion, and atrial and ventricular enlargement. Central pulmonary venous congestion and signs of early pulmonary edema also evident. [file 2859676.f1.zip › 2859676.f1/Image 6.jpg]

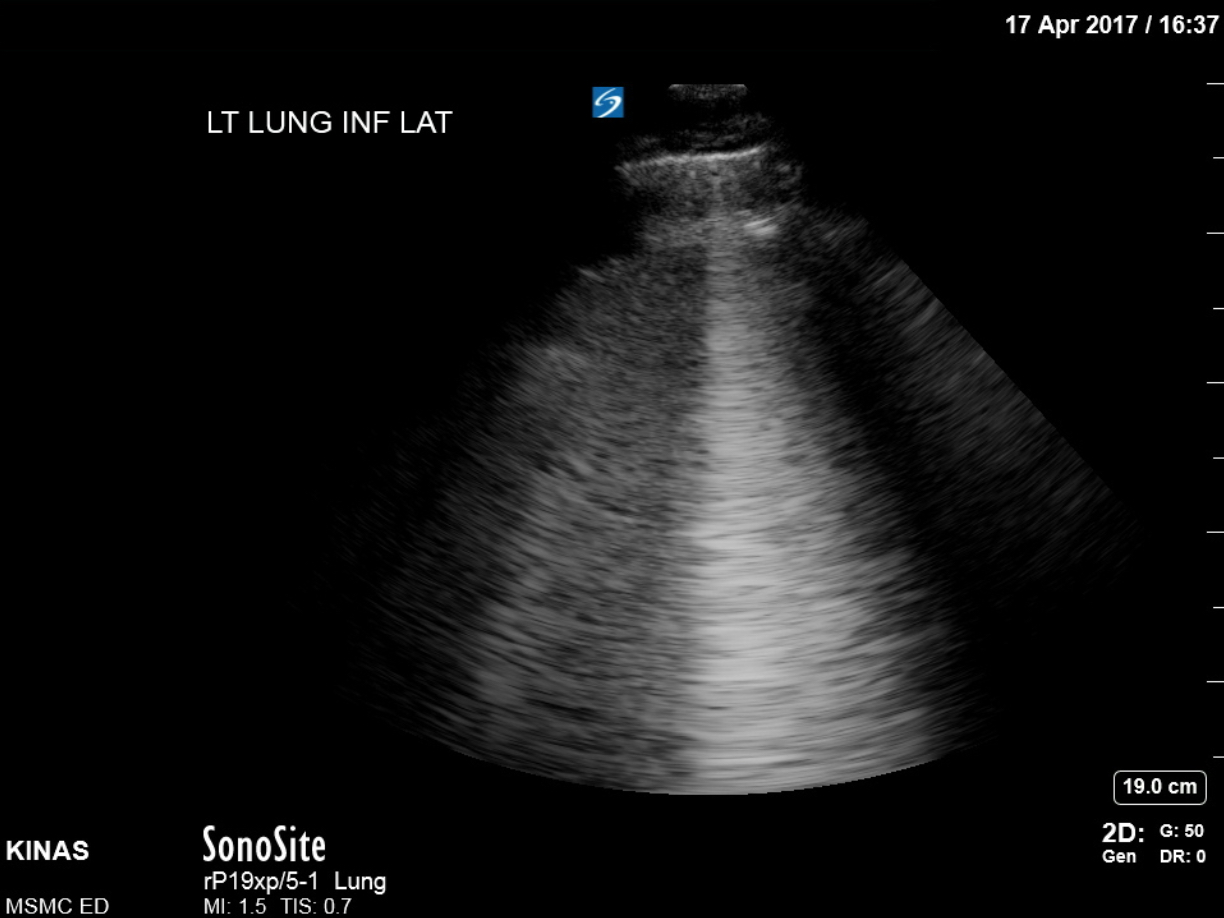

Supplement: Supplementary Materials — Image 1: ultrasound of long axis view of IVC in inspiration: maximal internal diameter of 1.71 cm. Image 2: ultrasound of long axis view of IVC in expiration: maximal internal diameter of 1.85 cm. Video 1: ultrasound long axis view of IVC: dilated with minimal respiratory variation. Image 3: ultrasound of parasternal long axis (PLAX) view of heart: biventricular dilation. Image 4: ultrasound of parasternal short axis (PSAX) view of heart: biventricular dilation and small pericardial effusion. Image 5: ultrasound of apical four chamber (A4C) view of heart view: biatrial and biventricular dilation. Video 2: ultrasound of apical four-chamber (A4C) view of heart view: biatrial and biventricular dilation with severely depressed systolic function. Video 3: ultrasound of parasternal short axis (PSAX) view of heart: biventricular dilation and small pericardial effusion. Image 6: lung ultrasound of right inferolateral lung field: prominent B-lines. Image 7: lung ultrasound of left inferolateral lung field: prominent B-lines. Video 4: lung ultrasound of right inferolateral lung field: prominent B-lines. Video 5: lung ultrasound of left inferolateral lung field: prominent B-lines. Image 8: ECG: sinus tachycardia. No evidence of pericarditis. Image 9: chest X-ray: right lower lobe consolidation with evidence of central pulmonary venous congestion and cephalization. Image 10: computed tomography (CT) pulmonary angiogram (angiography window): right lower lobe consolidation, and no evidence of pulmonary embolism. Image 11: computed tomography (CT) pulmonary angiogram (lung window): right lower lobe consolidation, small pericardial effusion, and atrial and ventricular enlargement. Central pulmonary venous congestion and signs of early pulmonary edema also evident. [file 2859676.f1.zip › 2859676.f1/Image 7.jpg]

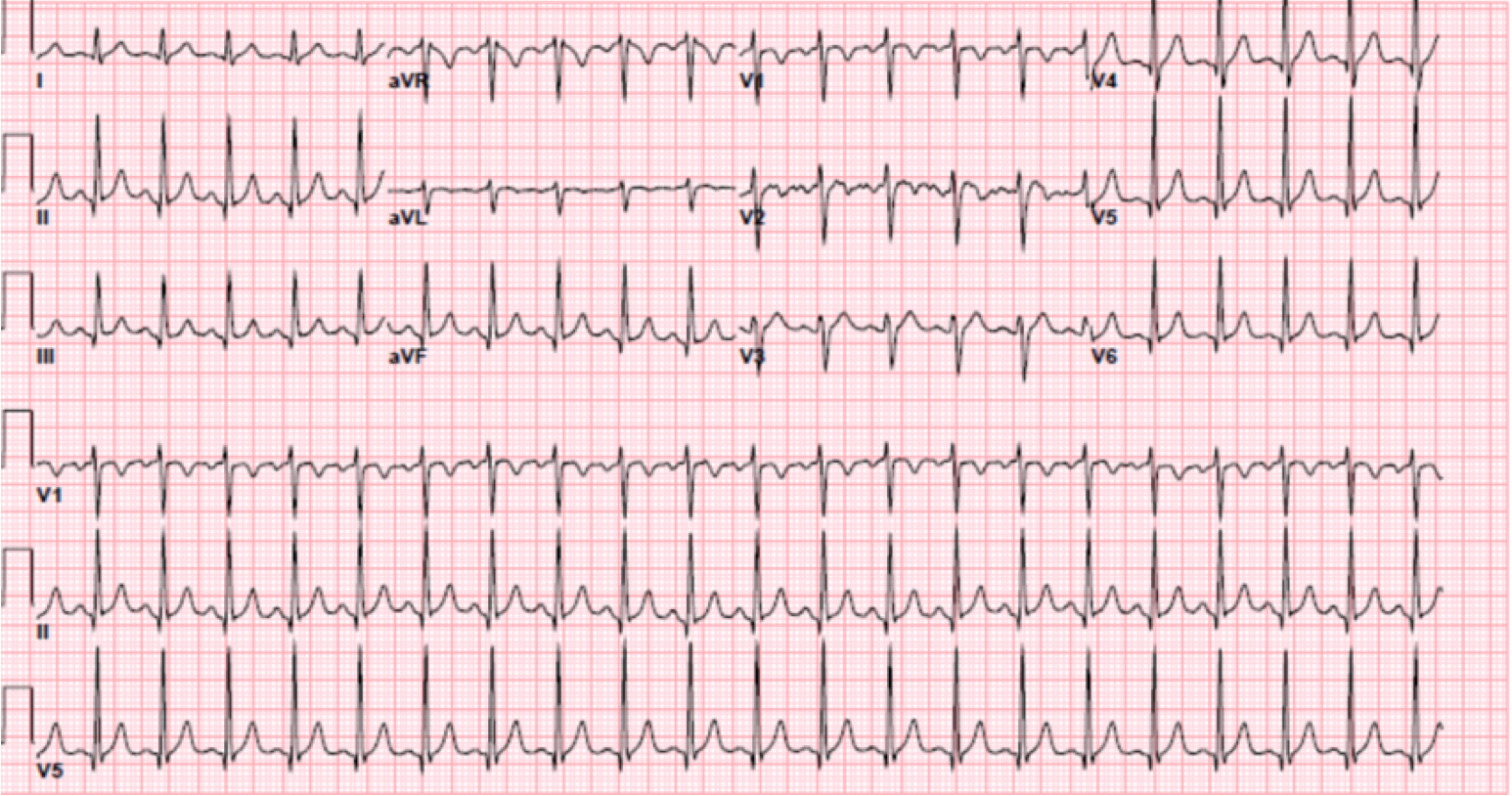

Supplement: Supplementary Materials — Image 1: ultrasound of long axis view of IVC in inspiration: maximal internal diameter of 1.71 cm. Image 2: ultrasound of long axis view of IVC in expiration: maximal internal diameter of 1.85 cm. Video 1: ultrasound long axis view of IVC: dilated with minimal respiratory variation. Image 3: ultrasound of parasternal long axis (PLAX) view of heart: biventricular dilation. Image 4: ultrasound of parasternal short axis (PSAX) view of heart: biventricular dilation and small pericardial effusion. Image 5: ultrasound of apical four chamber (A4C) view of heart view: biatrial and biventricular dilation. Video 2: ultrasound of apical four-chamber (A4C) view of heart view: biatrial and biventricular dilation with severely depressed systolic function. Video 3: ultrasound of parasternal short axis (PSAX) view of heart: biventricular dilation and small pericardial effusion. Image 6: lung ultrasound of right inferolateral lung field: prominent B-lines. Image 7: lung ultrasound of left inferolateral lung field: prominent B-lines. Video 4: lung ultrasound of right inferolateral lung field: prominent B-lines. Video 5: lung ultrasound of left inferolateral lung field: prominent B-lines. Image 8: ECG: sinus tachycardia. No evidence of pericarditis. Image 9: chest X-ray: right lower lobe consolidation with evidence of central pulmonary venous congestion and cephalization. Image 10: computed tomography (CT) pulmonary angiogram (angiography window): right lower lobe consolidation, and no evidence of pulmonary embolism. Image 11: computed tomography (CT) pulmonary angiogram (lung window): right lower lobe consolidation, small pericardial effusion, and atrial and ventricular enlargement. Central pulmonary venous congestion and signs of early pulmonary edema also evident. [file 2859676.f1.zip › 2859676.f1/Image 8.jpg]

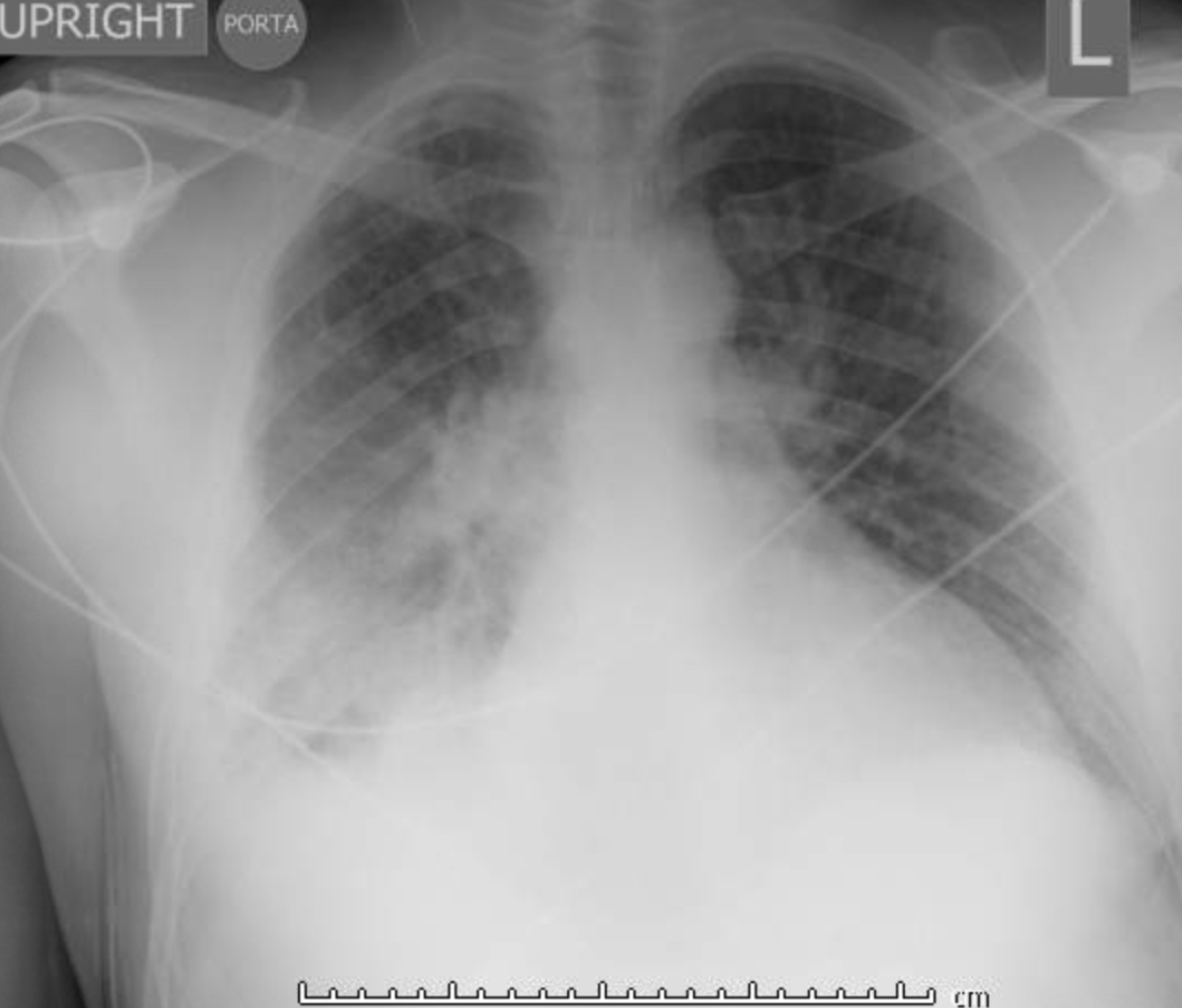

Supplement: Supplementary Materials — Image 1: ultrasound of long axis view of IVC in inspiration: maximal internal diameter of 1.71 cm. Image 2: ultrasound of long axis view of IVC in expiration: maximal internal diameter of 1.85 cm. Video 1: ultrasound long axis view of IVC: dilated with minimal respiratory variation. Image 3: ultrasound of parasternal long axis (PLAX) view of heart: biventricular dilation. Image 4: ultrasound of parasternal short axis (PSAX) view of heart: biventricular dilation and small pericardial effusion. Image 5: ultrasound of apical four chamber (A4C) view of heart view: biatrial and biventricular dilation. Video 2: ultrasound of apical four-chamber (A4C) view of heart view: biatrial and biventricular dilation with severely depressed systolic function. Video 3: ultrasound of parasternal short axis (PSAX) view of heart: biventricular dilation and small pericardial effusion. Image 6: lung ultrasound of right inferolateral lung field: prominent B-lines. Image 7: lung ultrasound of left inferolateral lung field: prominent B-lines. Video 4: lung ultrasound of right inferolateral lung field: prominent B-lines. Video 5: lung ultrasound of left inferolateral lung field: prominent B-lines. Image 8: ECG: sinus tachycardia. No evidence of pericarditis. Image 9: chest X-ray: right lower lobe consolidation with evidence of central pulmonary venous congestion and cephalization. Image 10: computed tomography (CT) pulmonary angiogram (angiography window): right lower lobe consolidation, and no evidence of pulmonary embolism. Image 11: computed tomography (CT) pulmonary angiogram (lung window): right lower lobe consolidation, small pericardial effusion, and atrial and ventricular enlargement. Central pulmonary venous congestion and signs of early pulmonary edema also evident. [file 2859676.f1.zip › 2859676.f1/Image 9.jpg]
